# An Even Bigger Win: Understanding the Neural Systems Underlying Motivational Influences on Ambiguous Social Perception

**DOI:** 10.3389/fnins.2012.00141

**Published:** 2012-09-28

**Authors:** Jennifer S. Beer

**Affiliations:** ^1^Department of Psychology, University of Texas at AustinAustin, TX, USA

As Leonardo Da Vinci's *Mona Lisa* illustrates, ambiguity can be fascinating. Is she smiling because she is contented? Amused? Knows a good secret? When the world presents us with ambiguous input, rather than a blind eye, we often strive all the more to interpret its meaning (Long and Toppino, 2004; Adolphs, 2006). Sometimes things are ambiguous because, without additional information, they are subject to multiple meanings. Other times, stimuli are ambiguous because they contain conflicting elements. What if Da Vinci had written “sad” across Mona Lisa's face, would your interpretation be initially dominated by the negative connotation of that label or the positive connotation of her smile? Is there anything really different about trying to inhibit your attention to the face compared to the label? A recent study suggests the label would be processed more quickly than her facial expression and that at least partially distinct brain systems are needed to inhibit the influence of a face compared to a word in a final judgment. Ovaysikia et al. (2011) presented participants with stimuli that depicted facial expressions of emotion overwritten with emotion words that varied in congruence with the facial expressions (e.g., congruent stimuli might depict a smiling face overwritten with the word “happy” whereas incongruent stimuli might depict a smiling face overwritten with the word “sad”). Participants processed words significantly more quickly than facial expressions. Furthermore, different subregions within the prefrontal cortex were recruited to suppress words compared to faces. We do not typically encounter faces with words on them (advertisements and the rare tattoo notwithstanding), so what is the significance of Ovaysikia et al.’s (2011) findings? The results challenge current theories about what kinds of information dominates as we try make meaning of ambiguous input. They also call attention to a number of important questions about meaning-making that have received very little attention in the neuroscience literature.

The findings challenge assumptions made by dual-process perspectives that currently dominate research on judgment in psychology, neuroscience, and economics. Dual-process perspectives suggest that we most typically make meaning (i.e., make a judgment) of ambiguous situations in a predictable, two-step process (Tversky and Kahneman, 1974). We first use automatic, instinctual processes to estimate a possible meaning and then adjust our instincts using learned processes which are theorized to most often be relatively controlled. The findings in Ovaysikia et al.’s (2011) study are evidence that meaning-making using “instinctual” processes is not always quicker than “learned” processes. The researchers note that interpreting facial expressions is arguably more instinctual than reading. Years before children learn to read, they spontaneously show interest in facial expressions compared to other objects (Mondloch et al., 1991) and can differentiate facial expressions of emotion for guidance in ambiguous situations (Stenberg, 2009). Therefore, one might expect that by adulthood, the long-term interest and experience with face perception would prioritize attention to faces in favor of words. In fact, previous research has found that faces can be prioritized over words (Beall and Herbert, 2008). Therefore, it was notable that participants in the Ovaysikia et al. (2011) study were quicker to respond and made fewer errors when asked to attend to the emotion words compared to when they were asked to attend to the emotional faces. The fact that sometimes the face wins (Beall and Herbert, 2008) and sometimes the words win (Ovaysikia et al., 2011) suggests that conceptualizations of instinctual processing should not necessarily be equated with automaticity.

The mixed findings about what “wins” in social perception also point to the importance of understanding how motivation and context influence interpretations of ambiguous social stimuli. Although research in other domains has begun to examine the influence of mindset on stimuli with conflicting meanings (e.g., Bhanji and Beer, in press), this approach is rarely adopted in current neuroscience research focused on understanding higher-order, social perceptions of faces or minds. Yet this perspective is important and ecologically valid. For example, the content of the emotion labels written across the emotional faces (Beall and Herbert, 2008; Ovaysikia et al., 2011) could just as easily be represented by individuals’ motivations, expectations, or contexts. What neural regions are recruited to imbue ambiguous social stimuli with meaning on the basis of motivation or context? Very little research has addressed that question. The potential is illustrated by investigations into the interpretation of an inherently ambiguous social cue, that is, surprised facial expressions. Amygdala activation is greatest when subjective interpretation or situational information lead to the conclusion that surprised facial expressions have arisen from unexpected, negative events (compared to unexpected, positive events: Kim et al., 2003, 2004).

A related question is how the brain supports control over the influence of expectations and context and whether this is different than control over relatively bottom-up aspects of social stimuli. The Ovaysikia et al. (2011) study concluded that controlling the processing of faces and words is not an equivocal task for the brain because they are not governed by a single neural system. The neural control of socioemotional processing has received quite a lot of attention (Heatherton and Wagner, 2011). As in the Ovaysikia et al. (2011) study, previous research has most frequently operationalized control as the need to inhibit initial or automatic attitudes or perceptions. However, social psychological models distinguish between inhibition and other kinds of control that may impact a social perception (Tversky and Kahneman, 1974). What is the relation between the neural regions associated with inhibiting the influence of an initial impression on judgment and the neural regions associated with adjusting initial impressions to account for new information? In one case, control is expressed to inhibit the influence of a particular factor whereas in the other case, control is needed to weigh several factors in a final judgment. Future research should probe situations in which adjustment is used to make meaning out of ambiguous stimuli.
